# Stablization of ACOs by NatB mediated N-terminal acetylation is required for ethylene homeostasis

**DOI:** 10.1186/s12870-021-03090-7

**Published:** 2021-07-03

**Authors:** Hai-qing Liu, Ya-jie Zou, Xiao-feng Li, Lei Wu, Guang-qin Guo

**Affiliations:** Institute of Cell Biology and MOE Key Laboratory of Cell Activities and Stress Adaptations, School of Life Sciences, Lanzhou University, Lanzhou, 730000 China

**Keywords:** Ethylene homeostasis, ACC oxidase, N-terminal acetylation, NatB

## Abstract

N*-*terminal acetylation (NTA) is a highly abundant protein modification catalyzed by N-terminal acetyltransferases (NATs) in eukaryotes. However, the plant NATs and their biological functions have been poorly explored. Here we reveal that loss of function of CKRC3 and NBC-1, the auxiliary subunit (Naa25) and catalytic subunit (Naa20) of Arabidopsis NatB, respectively, led to defects in skotomorphogenesis and triple responses of ethylene. Proteome profiling and WB test revealed that the 1-amincyclopropane-1-carboxylate oxidase (ACO, catalyzing the last step of ethylene biosynthesis pathway) activity was significantly down-regulated in *natb* mutants, leading to reduced endogenous ethylene content. The defective phenotypes could be fully rescued by application of exogenous ethylene, but less by its precursor ACC. The present results reveal a previously unknown regulation mechanism at the co-translational protein level for ethylene homeostasis, in which the NatB-mediated NTA of ACOs render them an intracellular stability to maintain ethylene homeostasis for normal growth and responses.

**Supplementary Information:**

The online version contains supplementary material available at 10.1186/s12870-021-03090-7.

## Introduction

N*-*terminal acetylation (NTA) is a form of a highly abundant co- or post-translational protein modification in eukaryotes, in which the α-amino group of a protein’s N-terminal amino acid is acetylated under the catalysis of N-terminal acetyltransferases (NATs) [1, 2]. NATs are classified as NatA-NatH based on substrate specificity and subunit compositions, which consist of at least one catalytic subunit and auxiliary subunit [3, 4]. The auxiliary subunits can function as a ribosome anchor, providing substrate specificity, and/or interaction with nascent polypeptides [5]. NTA can alter the steric or chemical properties of the modified N-terminus [6] that may affect protein-protein interaction [7], subcellular localization [8], stability or degradation necessary for normal cellular life in growth, development and responses. Although prevalent, the functional significance of NTA has only recently uncovered in plants, with a limited number of reports on its roles in embyogenesis [9], growth [10], flowering, reproduction [11], stress [12–16] and immunity to pathogens [17].

Ethylene is a classical plant hormone regulating growth and development. Although ethylene biosynthesis has been known as a relatively simple two-step pathway, in which ACO enzymes catalyze the production of ethylene in the last step from ACC, its regulation is still less well understood [18]. For decades it was believed that ACS is the rate-limiting enzyme of this pathway, promoting studies to investigate the regulation of ACS at different levels. However, emerging evidences in recent years reveal that ACO can also be rate-limiting in ethylene production under certain circumstances [19], implying that the ACOs may be also subjected to a stringent regulation, but its molecular mechanisms is so far barely known. The *Arabidopsis thaliana ACOs* genes have distinct tissue-specific expression patterns. For example, *ACO1* (*AT2G19590*) is highly expressed in embryogenesis [20]. *ACO2* (*AT1G62380*) is mostly expressed in the emerging seedling hypocotyl, where it is involved in the formation of the apical hook [21]. *ACO3* (*AT1G12010*) and *ACO5* (*AT177330*) are mainly expressed in the root [22], and *ACO4* (*AT1G05010*) is mostly expressed in vegetative tissue .

Here, we show that loss of function mutation of NatB led to reduced abundance of three of the five so far known functional ACOs (ACO2, ACO3, ACO4) to affect ethylene homeostasis for normal growth and responses. Thus the NatB-mediated NTA of ACOs render them an intracellular stability to maintain ethylene homeostasis in vivo.

## Results

### NatB is required for normal growth of Arabidopsis

Previously an Arabidopsis mutant named *ckrc3* caused by loss of function mutation of the gene encoding the auxiliary subunit (Naa25/TCU2) of a putative N^α^-terminal acetyltransferase B (NatB) was isolated as an auxin-deficient mutant in our lab. NatB is one of the eight Nats (NatA - NatH) found so far in eukaryotes, containing a catalytic (Naa20) and an auxiliary subunit (Naa25) to perform its full intracellular functions by co-translationally catalyzing the NTA of proteins beginning with MD/E/N/Q, as confirmed in our former experiments (paper under-revision elsewhere [23]) and others. Besides auxin-deficiency, *ckrc3* and *nbc-1* displayed pleiotropic developmental defects, including the significantly reduced growth rate (Supplemental Fig. S1), leaf reticulation, early flowering, aborted ovules in short silique (paper under-revision elsewhere).

### NatB depletion leads to down-regulation of ACO proteins

**Fig. 1:**
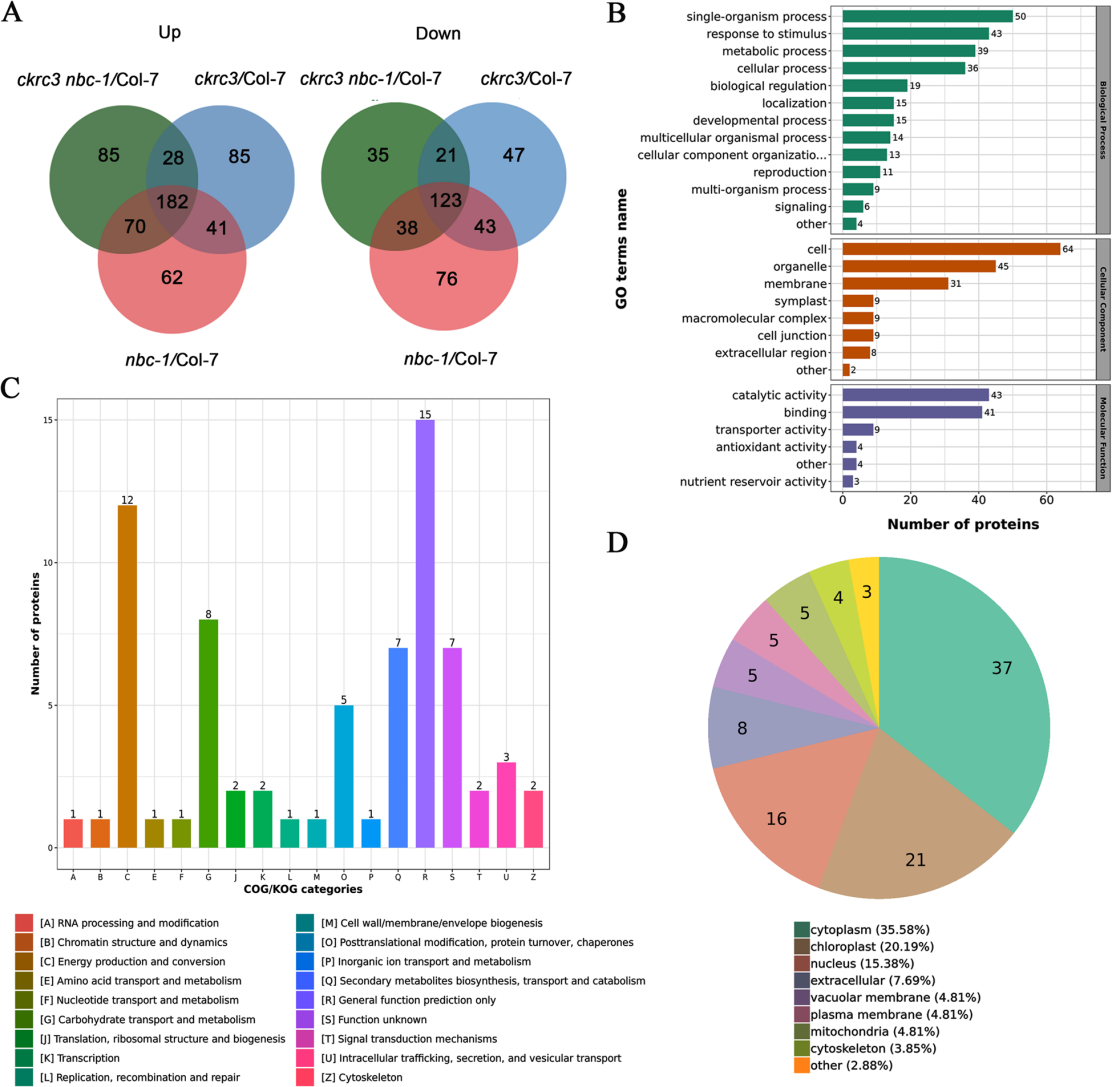
Proteome profiling. (**A**) Venn diagrams show up-regulated and down-regulated proteins. (**B**) Gene ontology (GO) analysis of differential protein based on the biological process (green bar), molecular function (brown bar) and cellular component (purple bar) categories. (**C**) Kyoto Encyclopedia of Genes and Genomes (KEGG) pathway annotation, differential expressed proteins played important roles in energy production and conversion, carbohydrate transport and metabolism, post-translation modification and secondary metabolites biosynthesis, transport and catabolism, according to KEGG enrichments. (**D**) Cluster analysis of subcellular localization showed the differential protein is mainly located in cytoplasm (35.58%), chloroplast (20.19%) and nucleus (15.38%)

Since NatB depletion would potentially modulate the abundance of many target proteins, we did global proteomics profiling in seedlings of *ckrc3*, *nbc-1*, and double mutant *ckrc3 nbc-1* and compared them with those in the wild type (Col-7) seedlings. A total of 6969 proteins were identified. 1.5-fold was taken as the change threshold of differential expression, and *p*-value < 0.05 was taken as the significance threshold of statistical t-test. In the *ckrc3 nbc-1*/Col comparison group, 365 protein expressions were up-regulated and 217 protein expressions were down-regulated (Fig. 1A), and differential expressed proteins were discovered to be involved in response to stimulus, post-translation modification and secondary metabolites biosynthesis, transport and catabolism and mainly located in cytoplasm according to GO classifications (Fig. 1B), KEGG enrichments (Fig. 1C) and cluster analysis (Fig. 1D).

**Fig. 2:**
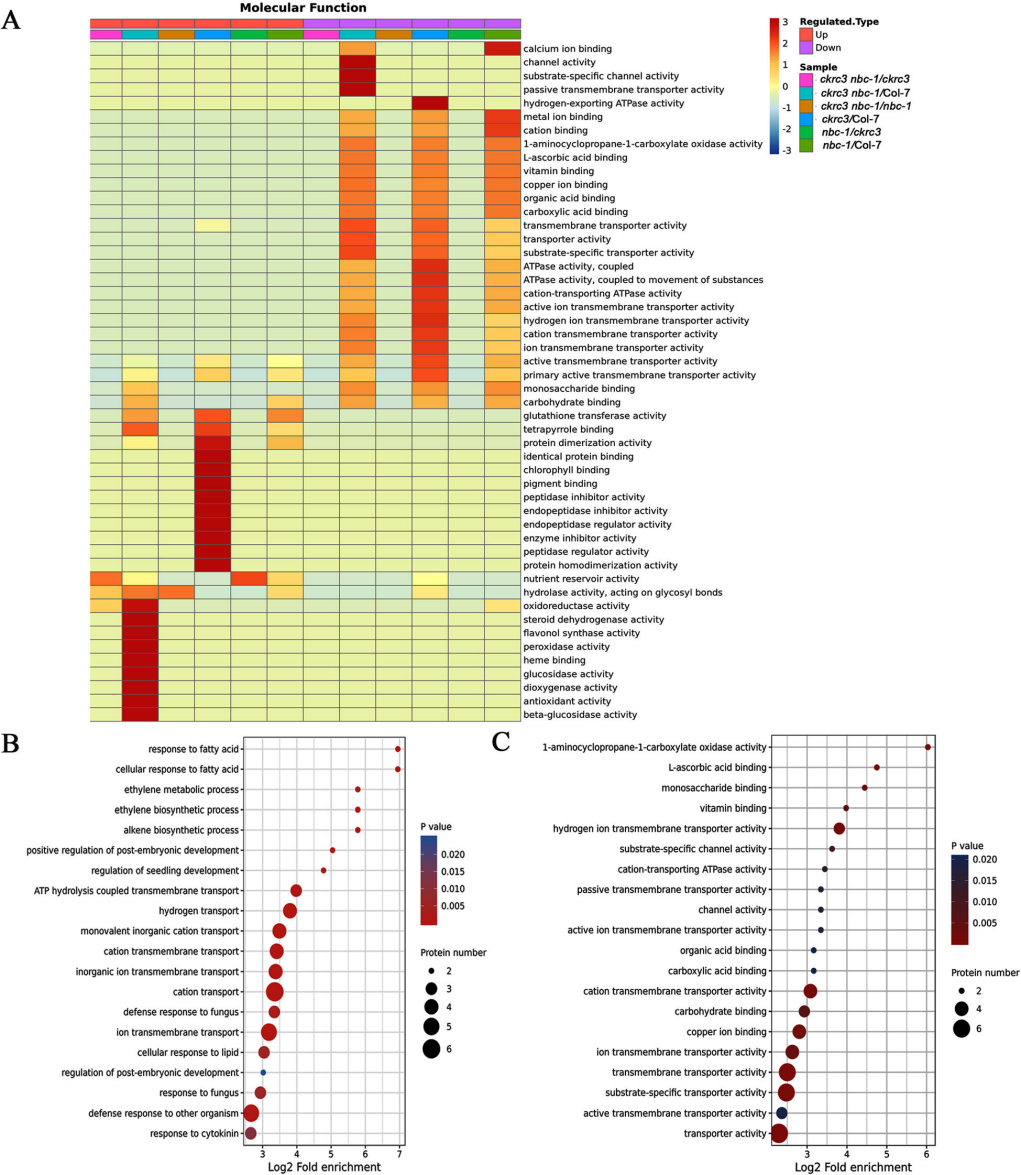
Identification of enrichment protein. (**A**) Cluster analysis heat map based on molecular function. Enrichment and distribution of differentially expressed proteins in GO functional classification bubble map (biological process, **C**) and (molecular function, **D**)

**Fig. 3:**
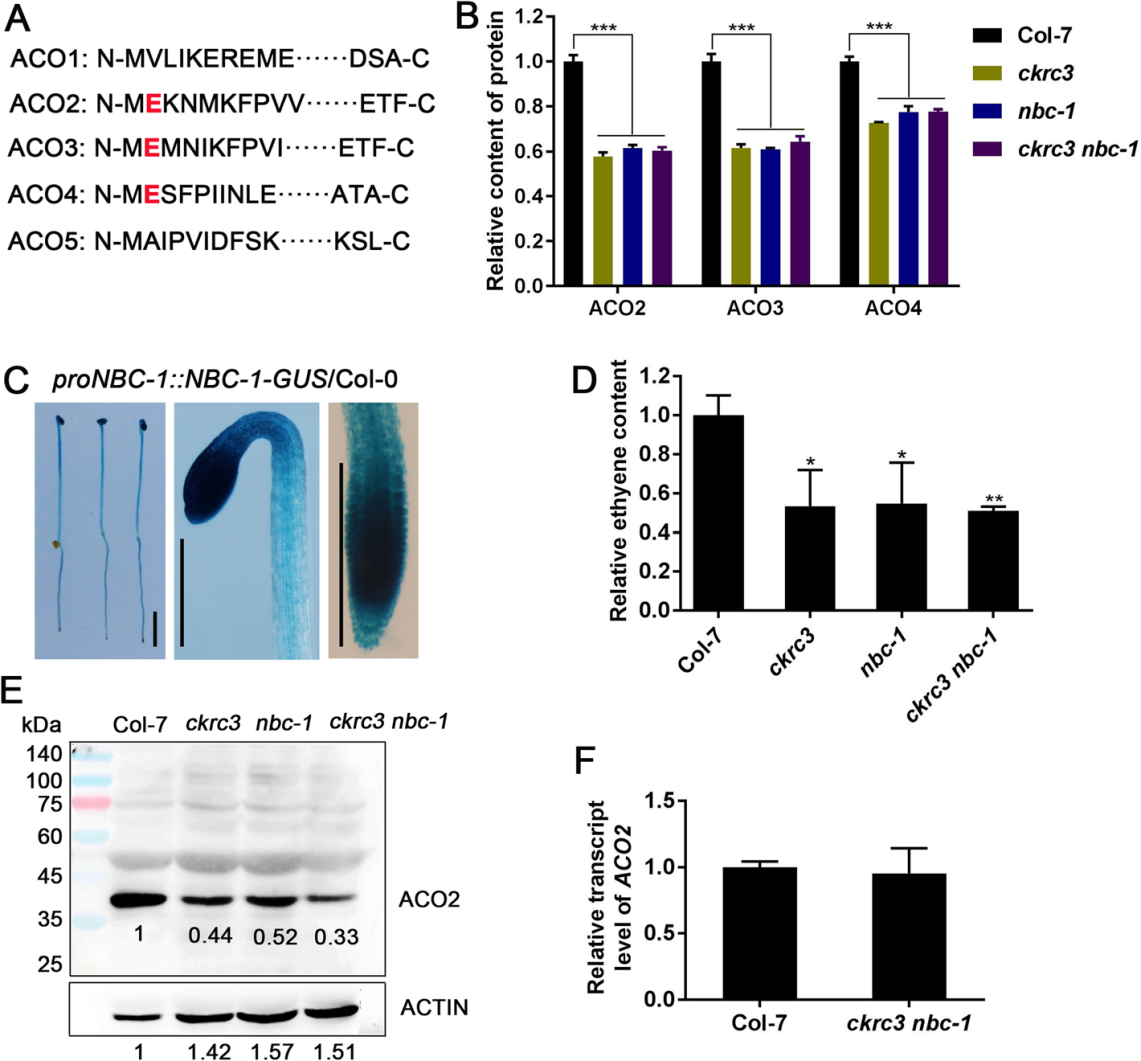
NTA mediated by NatB stabilizes ACOs. (**A**) N-terminal peptide of so far 5 known functional ACOs in ethylene biosynthesis in Arabidopsis, specific amino acid of NatB substrate signatures are shown in red colour. (**B**) Protein levels of ACO2, ACO3 and ACO4 extracted from the whole proteome analysis data. (**C**) GUS straining of *proNBC-1::NBC-1-GUS*/Col transgenic seedlings, bar = 2 mm. (**D**) Quantification results of ethylene contents. (**E**) ACO2 protein of dark-grown seedlings was detected using ACO2 antibody by WB test, the relative intensity of band in Col-7 was set to 1.0. (**F**) Transcript levels of *ACO2* in dark-grown Col-7 and *ckrc3 nbc-1* seedlings by RT-qPCR. “*”,“**” and “***” indicate significant differences according to ANOVA followed by Duncan’s multiple comparison, *p* < 0.05, *p* < 0.01 and *p* < 0.001, respectively

Protein annotation enrichment analysis revealed significant down-regulation of protein in a number of biological processes, including immune response, photosynthesis, regulation of proteolysis, regulation of defense response, negative regulation of hydrolase activity, response to host, ion transmembrane transport, hydrogen transport, and ethylene biosynthetic processes, etc. (Fig. 2A). Interestingly, we noted that ethylene biosynthetic processes were significantly down-regulated. In particular, several 1-amincyclopropane-1-carboxylate oxidases (ACOs) were remarkably down-regulated in the enrichment analysis of proteins based on biological processes and molecular function, (Fig. 2B-C, Fig. 3B).

### NTA catalyzed by NatB stabilizes ACO2

In *Arabidopsis*, three of the five so far known functional ACOs (ACO2, ACO3, ACO4) possess NatB substrate signatures (ME …, Fig. 3A). Their genes have distinct tissue-specific expression patterns. For example, *ACO2* (*AT1G62380*) is mostly expressed in the emerging seedling hypocotyl, where it is involved in the formation of the apical hook. We also detected relatively high expression levels of *NBC-1* in cotyledon, root, apical hook, hypocotyls in transgenic *proNBC-1::NBC-1-GUS* lines (Fig. 3C). The NatB-mediated NTA of ACO2/4 has just been reported to occur in Arabidopsis by acetylome analysis, which is significantly decreased in NatB-depleted mutants [10]. In our proteomics profiling data, ACO2/3/4 abundances were reduced 40, 36 and 30% in *ckrc3 nbc-1* compared with those in Col-7, respectively (Fig. 3B), which was also confirmed in WB experiment (Fig. 3E), whereas their transcription was not significantly different (Fig. 3F), indicating that the NTA of ACOs by NatB can stablize the protein post-transcriptionaly. These results are consistent with those of YUC8 in our another investigation [paper under revision elsewhere] as well as two new reports in which NatB-mediated NTA of SNC1 [17] and SIB1 [12] stabilize these two acetylated proteins in plants.

### *natb* mutants were insensitive to ACO inhibitor PZA

**Fig. 4:**
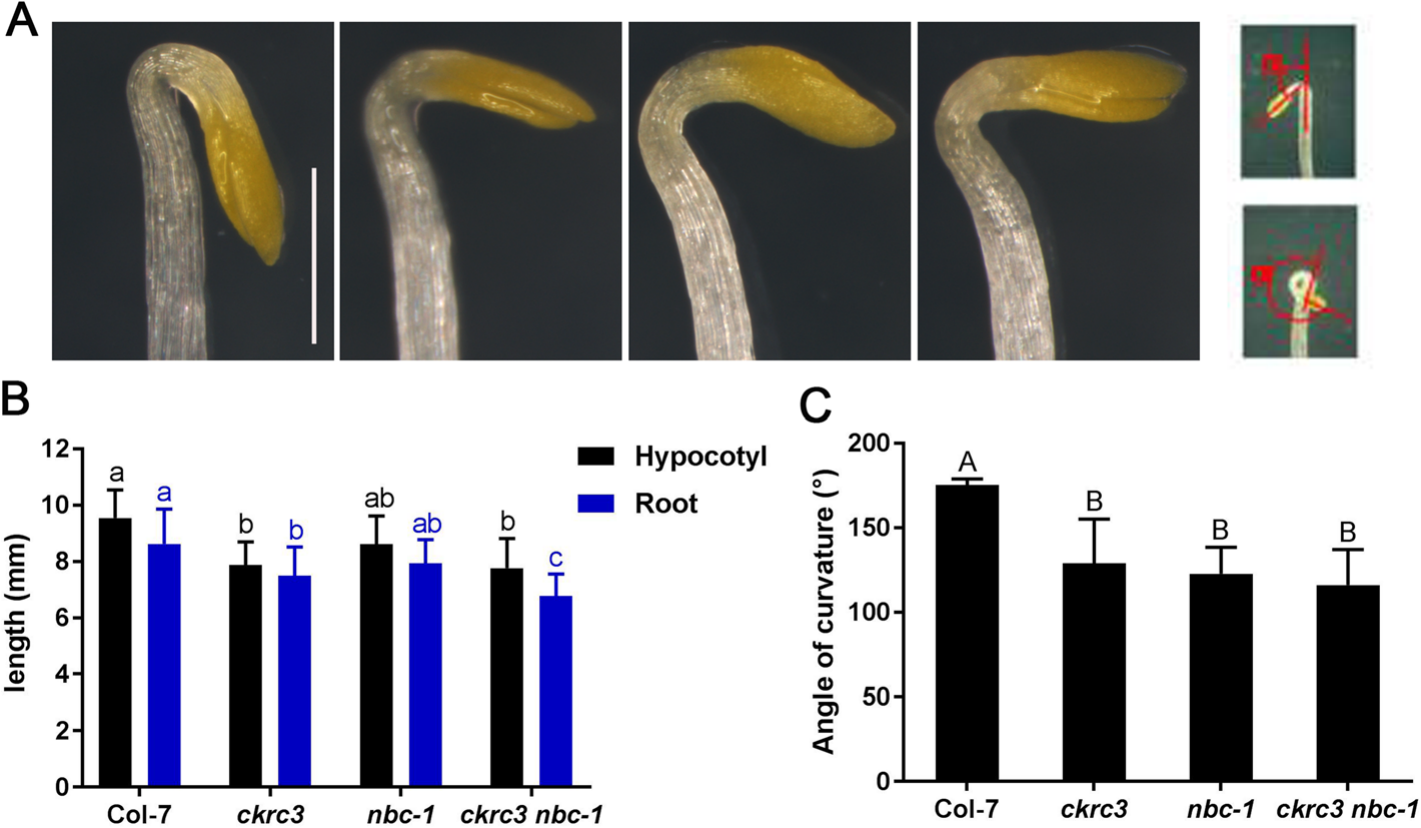
NatB mutant led to defects of skotomorphogenesis. (A) Phenotypes of apical hook of three DAG etiolated seedlings grown on MS, bar = 2 mm. (**B**) Quantification results of hypocotyls and roots length. (**C**) Quantification results of apical hook, different lowercase and capital letters above the bars indicate significant differences according to ANOVA followed by Duncan’s multiple comparison, *p* < 0.05, *p* < 0.001, respectively

**Fig. 5:**
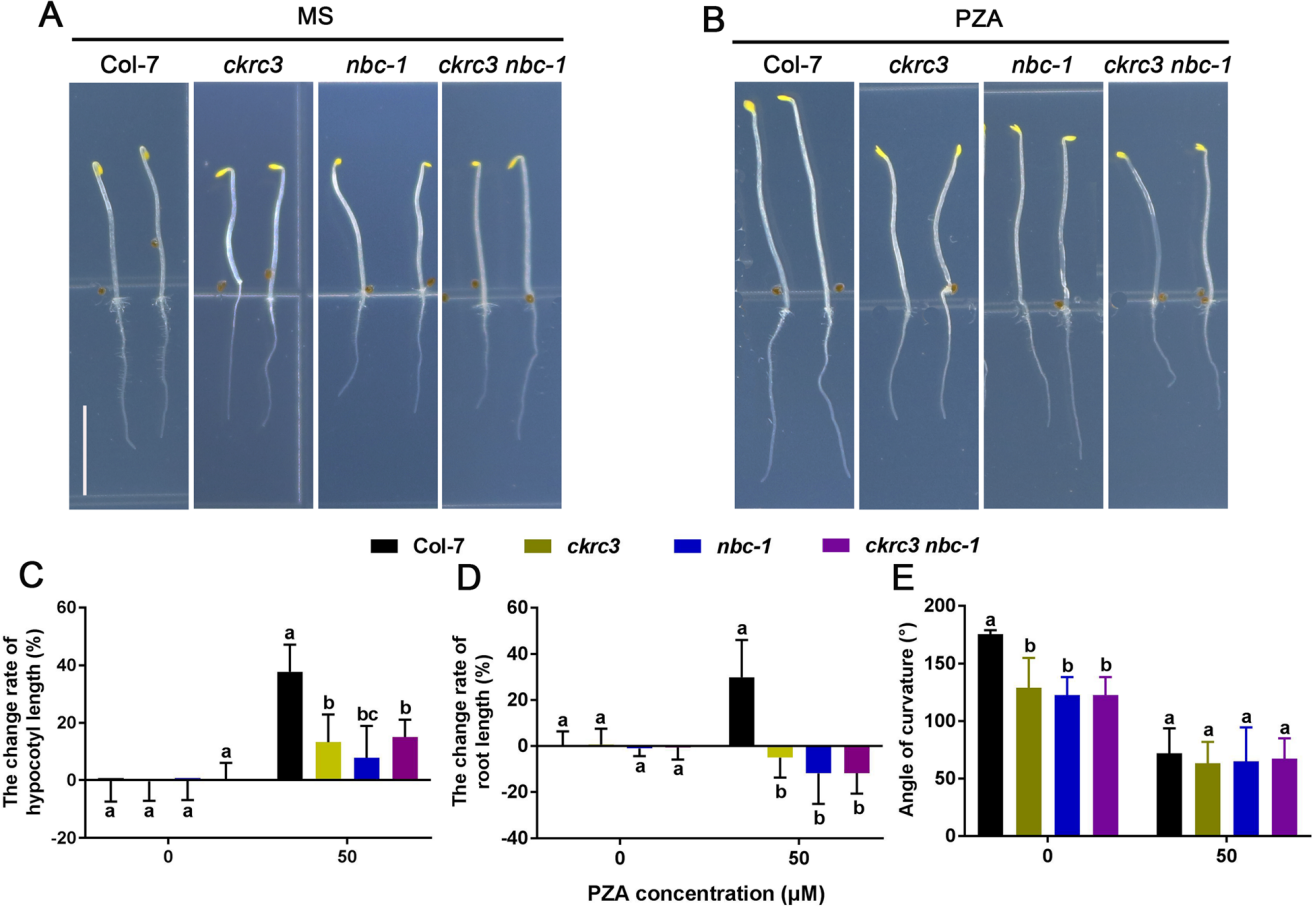
*natb* mutants were insensitive to ACO inhibitor PZA. (**A-B**) Phenotypes of three DAG (day after germination) etiolated seedlings grown on MS with or without 50 μΜ PZA, bar = 5 mm. (**C-E**) Quantification results of the change rate of hypocotyls length, root length, and apical hook. The change rate is 0 in 0 μΜ PZA, positive value means increase and negative value means decrease. Different lowercase letters above the bars indicate significant differences according to ANOVA followed by Duncan’s multiple comparison, *p* < 0.001

ACOs play an important role in skotomorphogenesis and ethylene response in Arabidopsis, so we examined whether NatB deficiency affects skotomorphogenesis, and finding that *ckrc3, nbc-1* and *ckrc3 nbc-1* showed defective skotomorphogenesis, including short hypocotyls and roots, and severe apical hook defects (Fig. 5A,Fig. 4). These results indicate that NatB is necessary for normal growth of Arabidopsis in darkness. To further demonstrate the effect of NatB-regulated ACOs on skotomorphogenesis was via ACOs-catalyzed ethylene biosynthesis, we treated etiolated seedlings with ACO inhibitor Pyrazinamide (PZA) [24], the wild type showed longer hypocotyls, roots and defective apical hooks (Fig. 5B-E), suggesting that PZA suppress the basal level of ethylene production, while, treatments of *natb* mutant with PZA slightly promoted the lengths of their hypocotyls and roots compared with wild type.

### NatB regulate ethylene homeostasis

**Fig. 6:**
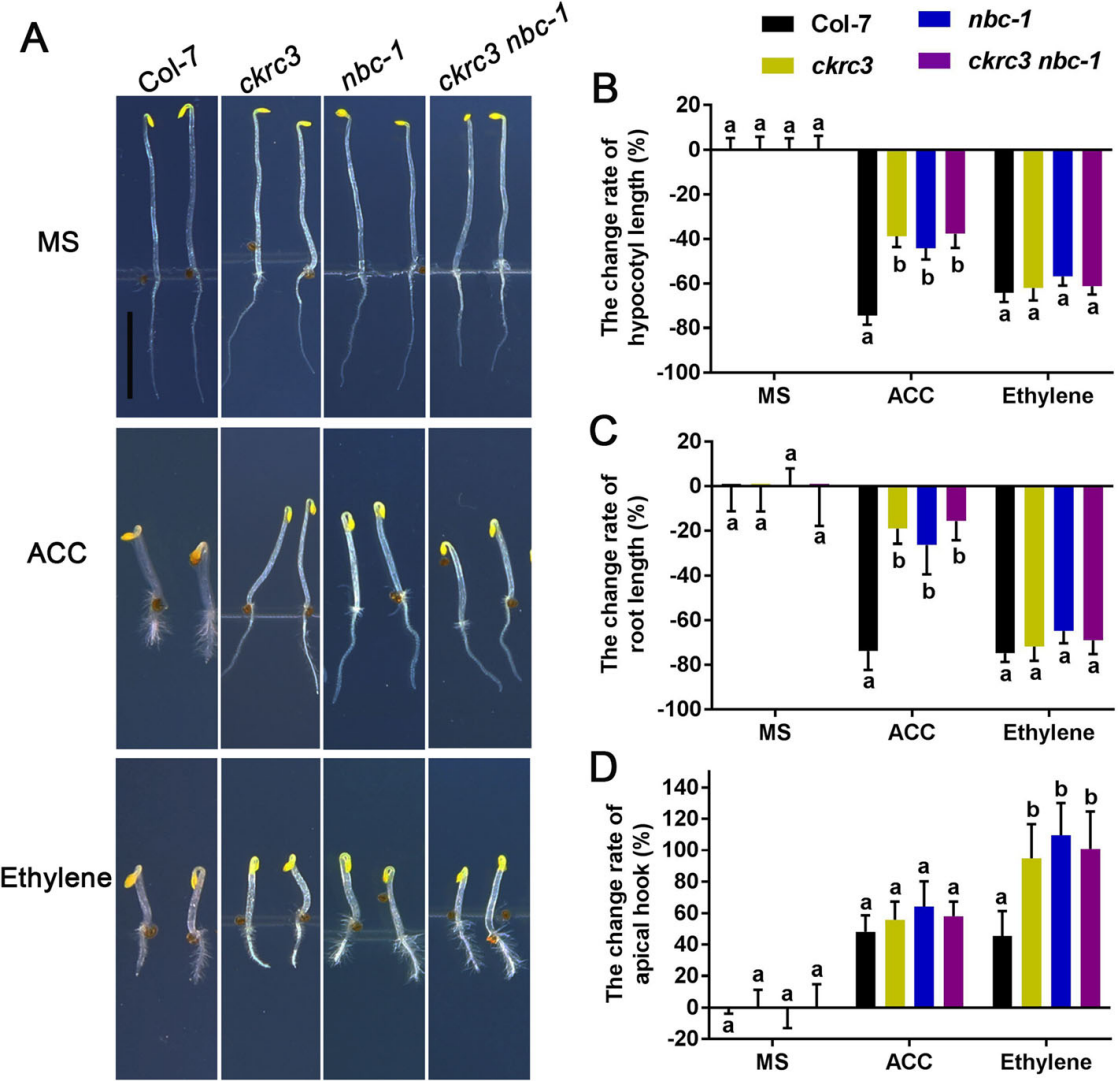
*natb* mutants could be fully rescued by application of exogenous ethylene, but less by its precursor ACC. (**A**) Phenotypes of three DAG etiolated seedlings grown on MS with or without 10 μΜ ACC / 15 ppm ethylene treatments, bar = 5 mm. (**B-D**) Quantification results of change rate of hypocotyls, root and apical hook. The change rate is 0 in MS, positive value means increase and negative value means decrease. Different lowercase letters above the bars indicate significant differences according to ANOVA followed by Duncan’s multiple comparison, *p* < 0.001

To directly confirm the role of NatB-mediated NTA of ACOs in ethylene homeostasis, we detected the ethylene content, finding a significant reduction in *ckrc3 nbc-1* mutant (Fig. 3D). Ethylene is required for proper skotomotogenesis and the triple-response in the dark, which play protecting roles for seedling before they grow out of soil. The low ethylene level caused by down-regulation of ACOs would led to defects in these processes. To check that, we observed the phenotypes of the 3 mutants in the darkness. As expected, defects in their triple responses (Fig. 6, Supplementary Fig. S2) were observed. Next, we examined the expression of two vital genes responsive to skotomotogenesis, *HLS1* and *ERF1,* detecting their significant down-regulations in *ckrc3 nbc-1* (Supplementary Fig. S3), confirming the involvement of NatB in regulating ethylene homeostasis and physiological responses. Notably, these ethylene-related phenotypes could be fully rescued by application of exogenous ethylene, but less by its precursor ACC (Fig. 6), consistent with the low steady-state levels of ACO2/3/4 in *NatB* mutants (Fig. 3B).

## Discussion

As one of the most common manner of protein modification in eukaryotes, N-terminal acetylation affects the molecular consequence of the substrates in several different ways, including protein stability, protein sorting, subcellular localization, protein folding, and protein interaction [1, 2, 6, 25]. NatB is presumed to N-terminal acetylate 15% of all yeast and 18% of all human proteins [3]. NatB deficiency will limit both the cellular and physiological function of different protein, and thus impacts a variety of biological processes. For instance, in yeast, the *naa20-Δ* and *naa25-Δ* deletion mutant strains display abnormal morphology, defects in mitochondrial division, and vacuolar segregation [26], it is also critical for meiosis, as it regulates the assembly of the synaptonemal complex in *Caenorhabditis elegans* [27]. The silencing of hNAA20 or hNAA25 in human cell lines induces growth arrest [28]. These studies reveal that NTA is involved in cellular processes in various organisms and is extremely important to higher eukaryotes.

In plants, the mutants of NatB subunits, *naa20* and *naa25*, exhibit several developmental defects. Loss of TCU2 function causes pleiotropic developmental defective phenotypes, including vegetative leaves that bend down to the primary vein, clustered flower appearance, shorter floral organ, shorter siliques [11]. Our study showed that NatB functions in normal growth of apical hook, hypocotyl and root of Arabidopsis (Fig. 4, Fig. 5A). Moreover, NatB has positive effect on ehthylene response by promoting ACO stability via NTA (Fig. 3). Xu et al. found that NatB depletion in Arabidopsis caused decreased immunity, which was caused by the destabilization of the plant immune receptor SNC1 [17]. NatB depletion also results in sensitivity to high-salt and osmotic stress [10, 29]. Most recently, NatB-catalyzed-NTA was reported to stabilize the stress/immune-related protein SIB1, enabling its prompt function and the related stress response [12]. These results indicate that in plants, NatB mediated protein NTA also plays vital functions in various processes of growth and responses.

Traditionally, ethylene, as a stress hormone, plays an important role in fruit ripening, tissue and organ aging. While ACO, as a rate-limiting enzyme in ethylene biosynthesis [30, 31], is mainly induced by stress factors, such as flood [32], oxygen deficit [33, 34], osmotic stress [35], and fruit ripening process [36, 37]. An increasing research suggests that ethylene plays an important role in seedling growth by crosstalk with auxin, gibberellin, brassinosteroids and other hormones [38–41], especially in apical hook formation, hypocotyl, and root growth [42–45]. Our study also shows that NTA of ACOs play an indispensable role in apical hook, hypocotyls, and roots growth by increasing their protein stability (Fig. 3); in contrast, their loss of function, with typical insensitivity to ACC but normal response to ethylene (Fig. 6), can cause severe growth defects. Therefore, we propose that the catalytic subunit NBC-1 and the auxiliary subunit CKRC3 function at the ribosome by forming a NatB complex, which actylates ME-startingN-termini directly in cytosol, by this way the NTA modification of ACOs render these proteins more stable to catalyze ethylene biosynthesis and maintain its homeostasis for the normal growth and responses in plants.

## Conclusion

In summary, our results reveal a previously unknown regulation mechanism at the co-translational protein level for ethylene homeostasis in which the NatB-mediated NTA of ACOs render them an intracellular stability to maintain ethylene homeostasis for normal growth and responses. In *Arabidopsis*, 3 of the 5 ACOs possess NatB substrate signatures (ME) but the others 2 of them not, catalyzing the last step of ethylene biosynthesis pathway in various tissue/organs throughout plant growth and development. As NatB is widely expressed throughout plant development, it would be necessary to investigate roles of NatB-mediated NTA of ACOs in the whole processes of plant growth and development, as well as in the response to stress etc.

## Material and methods

### Plant materials

The mutant *ckrc3* in the Col-7 background was isolated in our lab previous, and the mutant library was purchased from The European Arabidopsis Stock Centre, uNASC; http://arabidopsis.info/ (NASC ID: N31400). *nbc-1* in the Col-0 background, from NASC, and the double mutant *ckrc3 nbc-1* were obtained by crossing in our lab .

### Growth conditions and phenotype characterization

Seeds were sterilized with 0.1% HgCl_2_, thoroughly washed with d_2_H_2_O three times and placed on Murashige Skoog (MS) medium containing 1% agar and 1% sucrose. The plates were kept at 4 °C for 2 days in darkness, then transferred to white light for 8 h at 22 °C to stimulate germination, and subsequently to darkness at 20 °C for 3 days. For chemical treatment, 1-aminocyclopropanecarboxylic acid (ACC) and Pyrazinamide (PZA) was supplied to the MS medium for phenotypic analysis. For ethylene treatment, Sterilized seeds were sown in MS medium in 10 cm × 10 cm plates containing ethylene gas .

### RNA extraction and quantitative real-time PCR

RNA was isolated using Trizol (Invitrogen, http://www.invitro-gen.com/) and reverse-transcribed using a reverse transcription kit (Takara, http://www.takara-bio.com/). Quantitative RT-PCR was performed in Applied Biosystems real-time PCR equipment by using the TB green chemistry (Takara). *ACTIN8* was used as an internal control. Quantitative PCR analysis was performed with three different replicates for each biological sample. Three biological replicates were performed in each experiment.

### SDS-PAGE and Western blot

Protein samples were separated by SDS-PAGE and then were transferred to a nitrocellulose blotting membrane. After blocking with 5% bovine serum albumin, the film was incubated with primary antibody over night at 4 °C, then washed three times with TBST for 10 min and incubated with secondary antibody for 1 h at room temperature. After washing four times with TBST for 10 min, the film was illuminated using a luminous imaging system.

### Generation of ACO2 antibody

The purified synthetic peptide ATSLVEKDSEYPS of ACO2 protein was used as the antigen to immunize rabbits for antibody production, performed by Shanghai aibo Biotechnology Co., LTD, China (https://ablebio.company.lookchem.cn/).

### Proteomics profiling

The Arabidopsis seedling were grown on MS medium at 22 °C with a 16/8 h light/dark cycle for 10 day, About 0.5 g (3 independent biological replication) seedlings were extracted plant total protein with lysis buffer (8 M urea, 1% Triton-100, 10 mM dithiothreitol, and 1% Protease Inhibitor Cocktail). The protein was digested by trypsin at 1:50 trypsin-to-protein mass ratio. The tryptic peptides were desalted with Strata X C18 (Phenomenex) and TMT labeled. The tryptic peptides were fractionated into fractions by high pH reverse-phase HPLC using Thermo Betasil C18 column (5 μm particles, 10 mm ID, 250 mm length). Then Liquid chromatography (EASY-nLC 1000 UPLC system)-mass spectrometry (Q ExactiveTM Plus Thermo) analysis. The resulting MS/MS data were processed using Maxquant search engine (v.1.5.2.8). The whole proteomics test was completed by Hangzhou Jingjie Biology Co., LTD, China (http://www.ptm-biolab.com.cn/).

### Expression assay of the NBC-1 gene

A 3924-bp segment including the promoter and full-length genomic DNA of NBC-1 were amplified from Col-0 and cloned into pBI101 vector for generating *pNBC-1::NBC-1-GUS*/Col-0 transgenic lines. For GUS staining, 3-day-old seedling grown on MS medium in darkness were incubated in 1 mM X-gluc at 37 °C, the GUS staining were observed by using stereomicroscope.

### Ethylene quantification

100 mg seedlings of Arabidopsis grow 3 days in darkness were put into 5 ml chromatograph (GC) vials (Agilent) with 2 ml MS medium for 2 d in darkness. 0.5 ml of the headspace was taken from the vials, and was injected into an gas chromatograph equipment (GC-7820A). Chromatographic column: AT·Al_2_O_3_/S; Carrier gas: N_2_; Gas pressure: 18.33 Kpa; Splitless; Gasification chamber temperature: 200 °C.

### Statistical analysis

Data was expressed as mean ± SD. Statistical analysis were performed with analysis of variance (ANOVA) test to compare differences between more than two groups or treatments.

## Supplementary Information

**Additional file 1.**

**Additional file 2.**

## Data Availability

All the data supporting the results of this article are included within the paper and its supplementary file as figures or tables.
