# Supplementary material for: Stablization of ACOs by NatB mediated N-terminal acetylation is required for ethylene homeostasis
Source: BMC Plant Biol. 2021 Jul 3;21:320. doi: 10.1186/s12870-021-03090-7 (PMC8254318; doi:10.1186/s12870-021-03090-7)
Supplement: Supplementary file 1 — Additional file 1. [file 12870_2021_3090_MOESM1_ESM.docx]

**
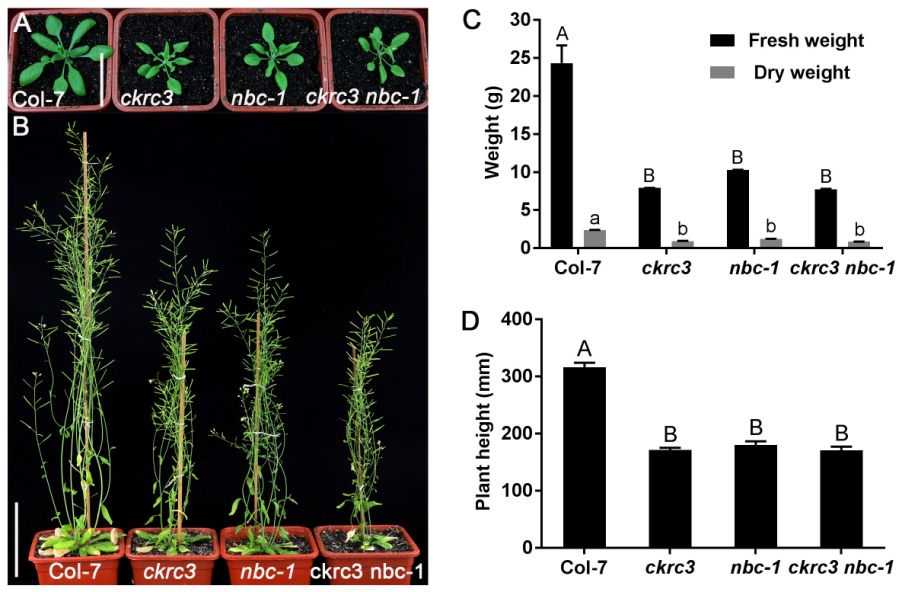
**

**Supplementary Fig S1**. NatB depletion results in reduced growth rate of seedlings. (**A**) Phenotypes of 30 day Col, *ckrc3*, *nbc-1*, *ckrc3 nbc-1* seedlings grown in soil, bar = 5 mm. (**B**) Phenotypes of 70 day Col, *ckrc3*, *nbc-1*, *ckrc3 nbc-1* seedlings grown in soil, bar = 5 cm. (**C**) Quantification results of fresh and dry weight of seedlings grown in soil for 30 day (n = 30 seedlings). (**D**) Quantification results of plant height. Different capital and low letters indicate significant differences at p < 0.001 according to ANOVA followed by Duncan’s multiple comparison tests.

**
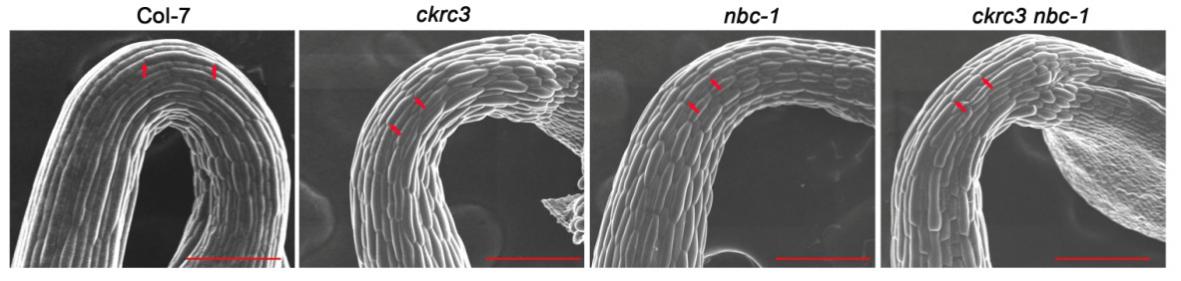
**

**Supplementary Fig S2**. Scanning electron microscopy of apical hook epidermal cells from 3 dag etiolated seedlings on MS medium, with one of the epidermal cells between the arrows in red, the cell on the convex side of mutant lacked sufficient elongation for normal hook development. Bar = 150 µm.

**
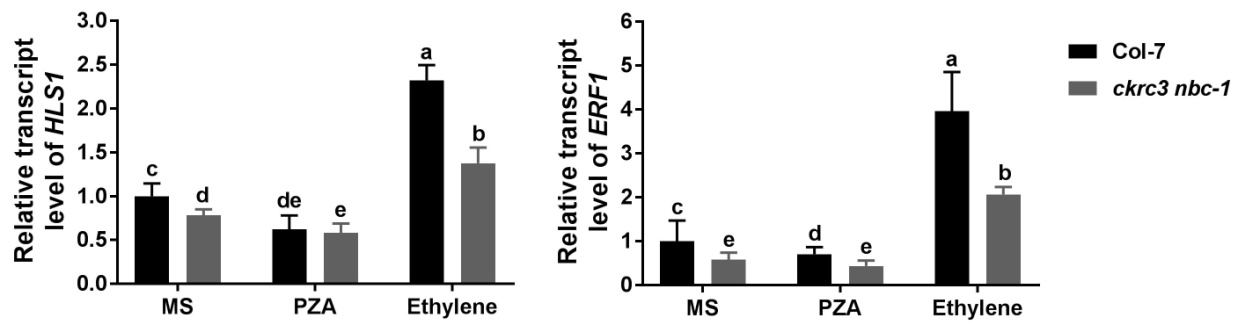
**

**Supplementary Fig S3**. The transcript levels of *HLS1* and *ERF1* in Col-7 and *ckrc3 nbc-1* etiolated seedlings grown with or without 50 μΜ PZA, 15 ppm ethylene. Different low letters indicate significant differences according to one-way ANOVA followed by Duncan’s multiple comparison, p < 0.001.
