# Supplementary figures and images for: Stablization of ACOs by NatB mediated N-terminal acetylation is required for ethylene homeostasis
Source: BMC Plant Biol. 2021 Jul 3;21:320. doi: 10.1186/s12870-021-03090-7 (PMC8254318; doi:10.1186/s12870-021-03090-7)

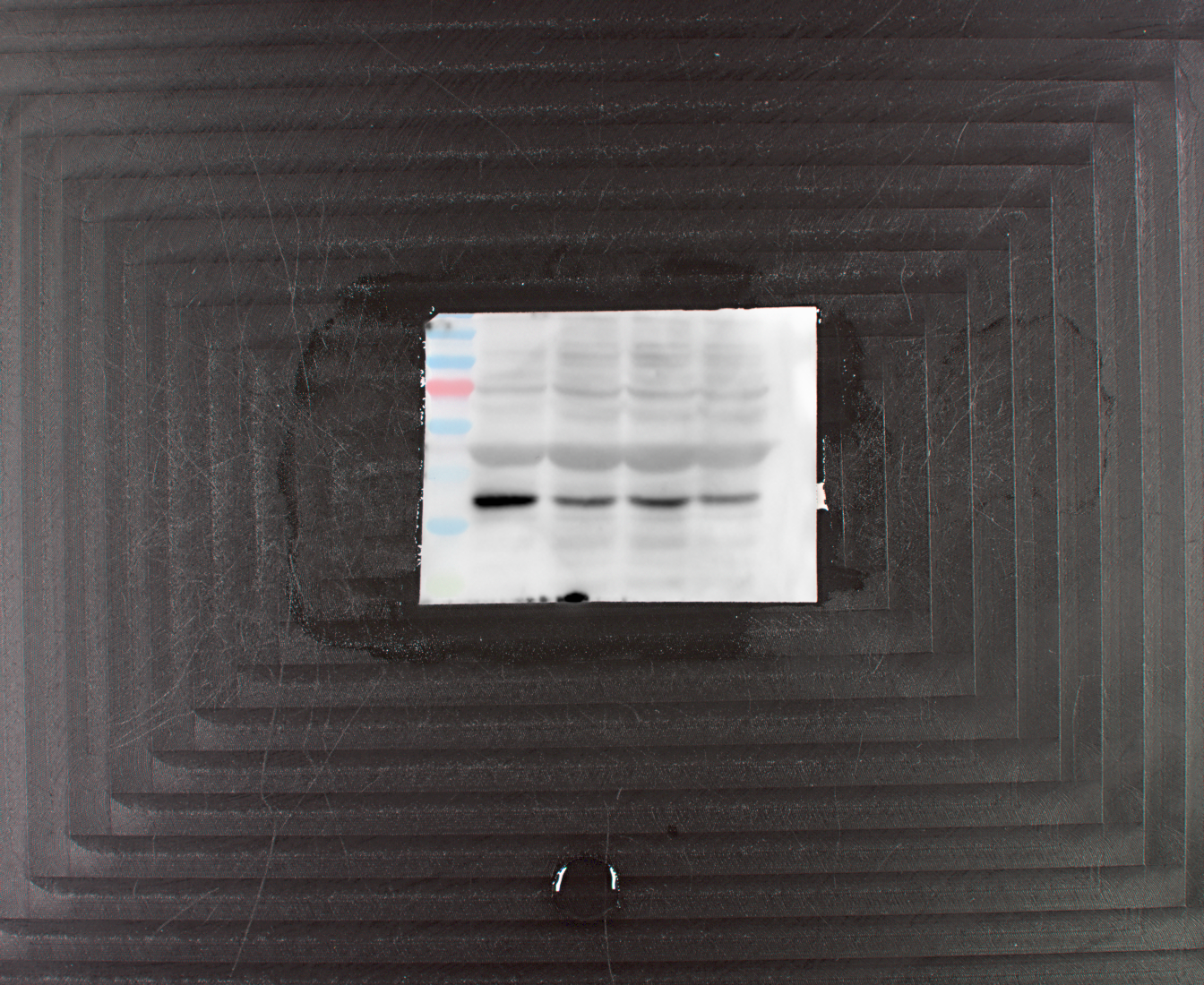

Supplement: Supplementary file 2 — Additional file 2. [file 12870_2021_3090_MOESM2_ESM.zip › additional files 2/WB-ACO2.BMP]

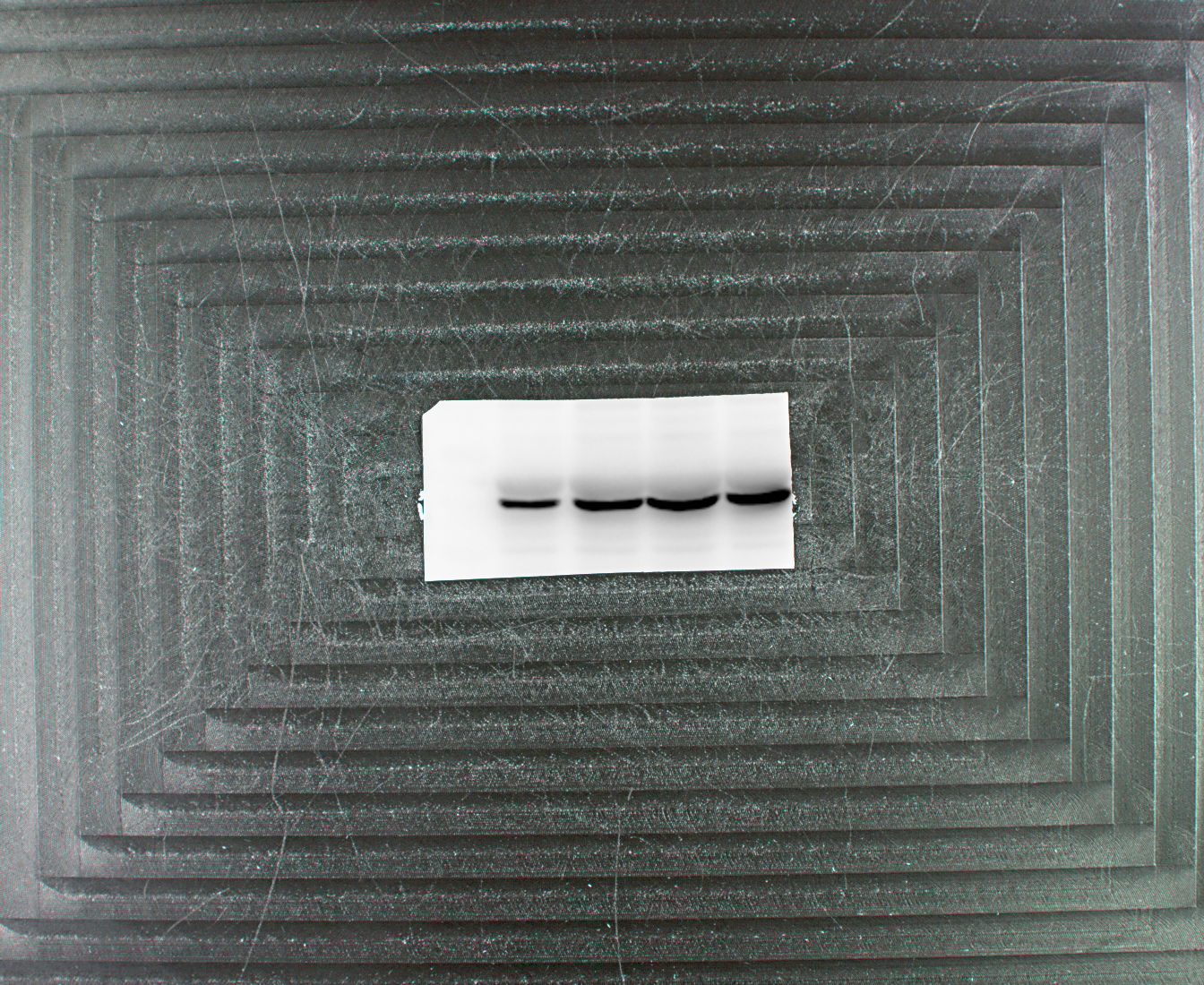

Supplement: Supplementary file 2 — Additional file 2. [file 12870_2021_3090_MOESM2_ESM.zip › additional files 2/WB-ACTIN.BMP]
